# RETRACTION: A Meta‐Analysis Examined the Effect of Stoma on Surgical Site Wound Infection in Colorectal Cancer

**DOI:** 10.1111/iwj.70469

**Published:** 2025-04-02

## Abstract

**RETRACTION:** ChangZ. , LiuL. , SheC. , RenW. , ChenH. , and ZhouC. , “A Meta‐Analysis Examined the Effect of Stoma on Surgical Site Wound Infection in Colorectal Cancer,” International Wound Journal 20, no. 5 (2024): 1578–1583, 10.1111/iwj.14013.PMC1008884236401595

The above article, published online on 19 November 2022, in Wiley Online Library (http://onlinelibrary.wiley.com/), has been retracted by agreement between the journal Editor in Chief, Professor Keith Harding; and John Wiley & Sons Ltd. Following an investigation by the publisher, all parties have concluded that this article was accepted solely on the basis of a compromised peer review process. The editors have therefore decided to retract the article. The authors did not respond to our notice regarding the retraction.

**RETRACTION:** Z. Chang , L. Liu , C. She , W. Ren , H. Chen , and C. Zhou , “A Meta‐Analysis Examined the Effect of Stoma on Surgical Site Wound Infection in Colorectal Cancer,” International Wound Journal 20, no. 5 (2024): 1578–1583, 10.1111/iwj.14013.PMC1008884236401595

The above article, published online on 19 November 2022, in Wiley Online Library (http://onlinelibrary.wiley.com/), has been retracted by agreement between the journal Editor in Chief, Professor Keith Harding; and John Wiley & Sons Ltd. Following an investigation by the publisher, all parties have concluded that this article was accepted solely on the basis of a compromised peer review process. The editors have therefore decided to retract the article. The authors did not respond to our notice regarding the retraction.

